# Association between Coalmine Dust and Mortality Risk of Lung Cancer: A Meta-Analysis

**DOI:** 10.1155/2021/6624799

**Published:** 2021-03-08

**Authors:** Linlin Li, Min Jiang, Xuelian Li, Baosen Zhou

**Affiliations:** ^1^Department of Clinical Epidemiology, First Affiliated Hospital, China Medical University, Shenyang 110001, China; ^2^Medical Oncology Department of Thoracic Cancer (2), Cancer Hospital of China Medical University, Liaoning Cancer Hospital & Institute, No. 44, Xiaoheyan Road, Dadong District, Shenyang, 110042 Liaoning Province, China; ^3^Department of Epidemiology, School of Public Health, China Medical University, Shenyang 110122, China

## Abstract

**Background:**

Evidence on the carcinogenicity of coalmine dust in occupational settings is still conflicting. Therefore, we conducted this research to evaluate the mortality risk of lung cancer for coalminers exposed to occupational dust when compared to population with no or low dust exposure.

**Methods:**

Databases of PubMed and Chinese National Knowledge Infrastructure as well as reference lists were searched updated to September 18, 2020. The enrolled articles should report lung cancer mortality risk for coalminers exposed to occupational dust. Basic information was extracted such as the author and publication year, area and ethnicity, the type and estimates of outcome, duration of follow-up, and the study design. The checklists from Agency for Healthcare Research and Quality and the Newcastle-Ottawa Scale were used for the assessment of quality and bias risk for descriptive studies, cohort studies, and case control studies, respectively. The overall relative risks were calculated while Begg's and Egger's tests and sensitivity analysis were performed to explore potential heterogeneity sources. Metaregression and subgroup analyses were also conducted to give more detailed information for the correlation between dust exposure and lung cancer mortality.

**Results:**

A total of 19 articles with 22 different studies (descriptive study, case control study, and cohort study) including 8909 observed deaths from 1964 to 2017 were enrolled with a significant heterogeneity (*I*^2^ = 95%, *P* < 0.001). The pooled relative risk of mortality from lung cancer was 1.16 (1.03-1.30) for coalminers. Results of metaregression analysis indicated that the high heterogeneity among these enrolled studies might be caused by the ethnicity differences (*P* = 0.011). Subgroup analysis also indicated that the pooled estimate for Asian population in China was 4.94 (3.95-6.17) with *I*^2^ = 39.3% and *P* = 0.192. All these results suggested that exposure to occupational dust would significantly increase the mortality risk of lung cancer, especially for Asian population in China, which should be measured and controlled more strictly. *Discussion*. This systematic review and meta-analysis provides high-quality evidence that exposure to occupational dust might increase the mortality risk of lung cancer, especially for Asian populations in China. The magnitude of this effect is of major public health importance in view of the ubiquitous existence of coalmining industry in China and even in the world. However, these pooled estimates should be interpreted cautiously because of the high heterogeneity among these publications. *Other*. This study was supported by the National Key Research and Development Program of China (Grant No. 2016YFC1302501).

## 1. Introduction

There is an increasing urgency in estimating the burdens of different diseases, especially of different cancers [1, 2], which can help to identify high-risk populations and the risk factors and take priority actions for risk reduction and health improvement.

Coalminers tend to be exposed to a series of risk factors during work such as mine dust, vibrations transmitted to the hands, noise, and a high heat load as well as an overloading of the upper extremities when working with pneumatic tools. Among these risk factors, mine dust containing crystalline forms of silica might be the most serious one that can significantly affect the health and life of coalminers.

In recent years, death from respiratory diseases is still an important occupational hazard for coalminers. The relationships between occupational exposures to coalmine dust and mortality from coal workers' pneumoconiosis and chronic obstructive pulmonary disease have been clearly established [3–5]. The mortality risk of lung cancer for coalminers has also been assessed in a series of epidemiological studies [6–8]. The working group of the International Agency for Research on Cancer (IARC) evaluated the carcinogenicity of coal dust by reviewing these epidemiological reports, but no clear conclusion was published [9].

Therefore, this current work was conducted to evaluate the mortality risk of lung cancer for coalminers exposed to occupational dust when compared to population with no or low dust exposure, which can facilitate the development of coalminers' risk reduction and health management. In addition, we also aimed to explore the differences of mortality risk of lung cancer for coalminers from different ethnicities, study designs, and outcome types based on the subgroup analyses.

## 2. Materials and Methods

### 2.1. Search Strategy

This analysis was reported based on the Preferred Reporting Items for Systematic Reviews and Meta-Analyses (PRISMA) statement and checklist. Both PubMed and Chinese National Knowledge Infrastructure (CNKI) databases were searched for relevant publications. The full search strategy without any limits for these two databases was as follows: (coal mine or coal mining) and (lung tumor or lung carcinoma or lung neoplasm or lung cancer) and dust, updated to September 18, 2020. Reference lists of the enrolled publications were also retrieved for acceptable literatures.

### 2.2. Publication Selection and Data Extraction

A series of criteria were drawn up for the inclusion and exclusion of publications, which were in accordance with the goal of this study. For the literature inclusion, the criteria were as follows: (1) target populations enrolled in the studies were coalminers exposed to occupational dust while the compared participants were individuals with no or low dust exposure; (2) lung cancer mortality should be reported as an outcome; (3) the original estimates of mortality along with 95% confidence intervals (95% CI) in the form of standardized mortality ratio (SMR), proportional mortality ratio (PMR), odds ratio (OR), or relative risk (RR) were reported in the articles; (4) the enrolled studies should be descriptive studies, case control studies, or cohort studies. For the exclusion, the criteria were as follows: (1) unrelated publications, reviews, case reports, and comments; (2) articles shared the same cohort or population; and (3) estimates of outcomes and the 95% confidence intervals as well as other information that are needed by the synthesized meta-analysis were missing. For studies including a wider population than our inclusion criteria, the eligible participants would be enrolled in our analysis while ineligible participants would be excluded. Overall, the process for determining which studies were eligible for inclusion could be concluded as publication search, exclusion for reviews, case reports, comments, unrelated and duplicated articles as well as articles without complete information through the full text scanning, and then the final remaining publications would be enrolled in this meta-analysis.

The information that was needed during the meta-analysis at both study level and participant level was extracted from the included publications by two authors independently as listed: the first author, publication year, participants' age, gender, area and ethnicity, number of observed deaths, the type and estimates of outcome with 95% CI, duration of follow-up, type of control and case groups, and the study design. Moreover, the duration of follow-up from different publications was unified by year, while the estimates of outcome were standardized in the form of mortality ratio.

### 2.3. Assessment for Study Quality and Risk of Bias

The methodological quality of the descriptive studies was assessed using the 11-item checklist recommended by the Agency for Healthcare Research and Quality (AHRQ), which included the definition of information source, inclusion and exclusion criteria, time period and continuity for identifying patients, blinding of personnel, assessments for quality assurance, confounding and missing data, and patient response rates and completeness, while the Newcastle-Ottawa Scale (NOS) was followed for the assessment of quality and bias risk for cohort studies and case control studies. For case control study, the items referred to the definition, selection and representativeness of cases and controls, comparability of cases and controls, ascertainment of exposure, and nonresponse rate, while for cohort study, selection and representativeness of the cohorts, ascertainment of exposure, assessment of outcome during the study, comparability of cohorts, and adequacy of follow-up were the major assessment items. An item would be scored “0” if it was answered “UNCLEAR” or “NO”; for the answer of “YES”, the item would get a “1” score. Article quality for descriptive study was assessed as follows: low quality: 0-3; moderate quality: 4-7; and high quality: 8-11. The score on the NOS ranged from 0 to 9 with a score equal to or greater than 6 identified as a feasible methodological design. For study with low quality or significant risk of bias, it would be eliminated from the following data synthesis.

### 2.4. Statistical Analysis

Lung cancer mortality risk was prespecified as the primary outcome in this study, which was compared between coalminers exposed to occupational dust and general population without dust exposure or occupational population with low dust exposure in the form of SMR, PMR, OR, or RR with their 95% CIs. This study used a two-stage approach that first generated estimates of effectiveness for each study separately and then combined these summary statistics, which can be shown from the forest plots. The heterogeneity among these enrolled studies was assessed by *Q* test and *I*^2^ value [10]. *P* value for the *Q* test less than 0.05 or *I*^2^ > 50% indicated that there was significant heterogeneity, and then, the overall estimate with 95% CI was pooled with the random-effect models. Otherwise, we would conduct the data synthesis with the fixed-effect models. In order to avoid the bias in the process of data synthesis caused by the fact that studies with large samples and positive results were easier to be published and retrieved than studies with small samples or negative results, Begg's and Egger's tests were performed to assess the existence of publication bias in this study, which would not be considered existed when the points representing the literature were distributed symmetrically or uniformly on both sides of the midline and mainly concentrated at the funnel tip and *P* > 0.05 for the Egger test. In addition, the sensitivity analysis was carried out by excluding the literature one by one. If the pooled estimates and 95% confidence intervals were evenly distributed on both sides of the midline and closely arranged, we can conclude that the research was stable and reliable; otherwise, the literatures deviating from the midline seriously should be excluded to improve the quality of the meta-analysis. Finally, subgroup analyses and metaregression analysis were conducted to identify potential sources of the heterogeneity, which were grouped by type of outcome: SMR, PMR, OR, and RR; ethnicity: Asian, Caucasian, and Caucasian/Negro; control group: general population, coalminers, and quarrymen; case group: coalminers or coalminers with pneumoconiosis; type of study design: cohort study, case control study, and descriptive study; number of observed deaths: <100 and >100; year of publication: 1964-1980, 1980-2000, and 2000-2017; duration of follow-up: <10 years, >10 years and <20 years, and >20 years; area: China, UK, US, and others; and study quality: moderate quality and high quality. All statistical analyses were performed by STATA 11.0 (StataCorp, College Station, TX, USA), and results of *P* < 0.05 were considered as statistically significant.

## 3. Results

### 3.1. Publication Search and Studies' Characteristics

**Figure 1:**
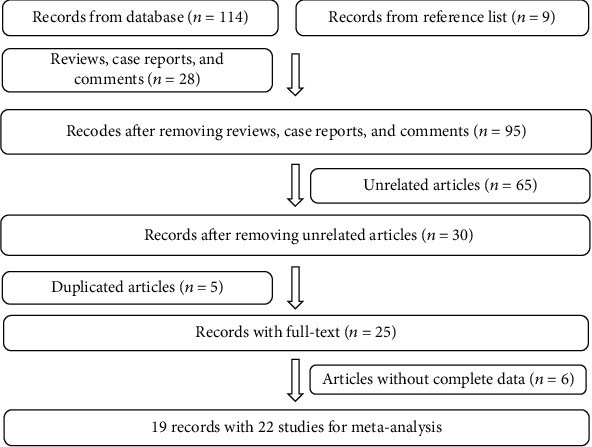
Flow diagram of study selection.

As shown in Figure 1, according to the primary protocol of the publication search, 123 eligible articles were enrolled, of which 28 articles were excluded as reviews, case reports, and comments. And then, 65 unrelated articles and 5 duplicated articles were excluded, leaving 25 papers with full texts. Further, another 6 articles were removed due to incomplete information. Ultimately, we obtained 19 articles with 22 different studies from 1964 to 2017 [6–8, 11–26]. The main characteristics of these 22 studies are listed in Table 1. The study quality of the enrolled articles was generally moderate and high with low risk of bias as shown in Supplementary Materials (Tables S1–3).

### 3.2. Pooled Analysis

**Figure 2:**
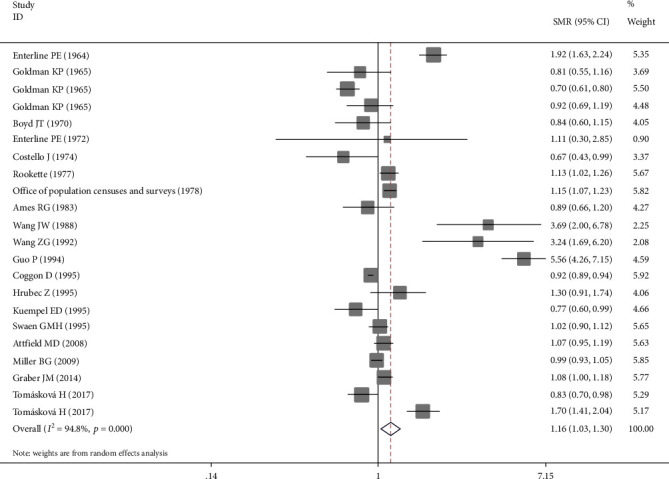
Forest plot of the pooled risk estimate for the association between exposure to coalmine dust and mortality risk from lung cancer.

The random-effect model was performed since significant heterogeneity existed among these 22 studies (*I*^2^ = 95%, *P* < 0.001). The pooled mortality estimate was 1.16 (95% CI: 1.03-1.30) with *P* = 0.013 as shown in Figure 2, which indicated that coalminers exposed to occupational dust might be at an increased mortality risk from lung cancer.

### 3.3. Publication Bias and Sensitivity Analysis

**Figure 3:**
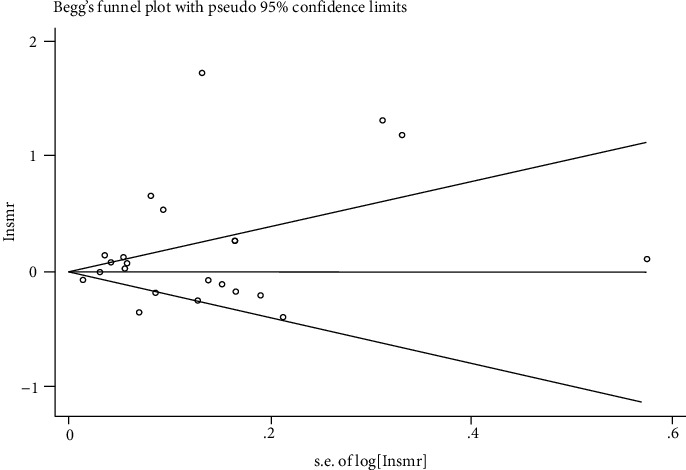
Begg's funnel plot to assess the publication bias for the enrolled studies.

According to Begg's funnel plot and Egger's regression test, it was found that the dots representing the literatures in Begg's funnel plot were uniformly distributed between the upper and lower ends of the middle line of the funnel plot and were symmetrical and concentrated at the tip of the funnel, while the *P* value of Egger's test was 0.089, indicating that there was no publication bias in the literatures included in this meta-analysis (Figure 3).

**Figure 4:**
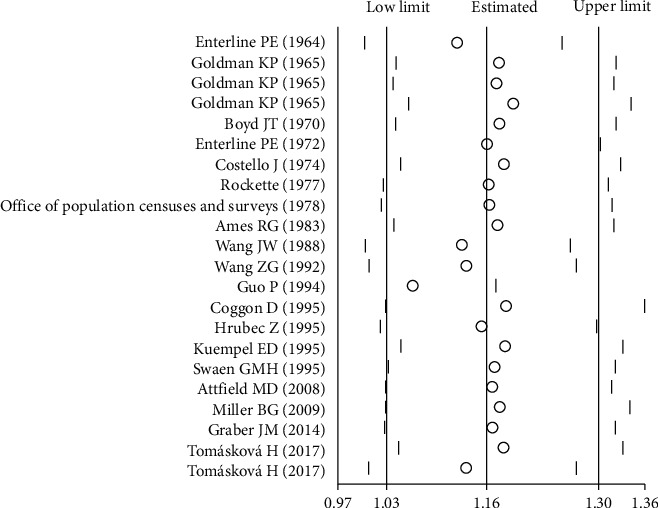
Sensitivity analysis for the association between exposure to coalmine dust and mortality risk from lung cancer.

The sensitivity analysis was also performed by excluding the publications one by one from the included literatures to assess the impact of each study data on the combined effect size. As shown in Figure 4, the result of the sensitivity analysis showed that the combined estimated values were evenly and closely distributed on both sides of the midline, indicating that each study had no statistically significant impact on the combined effect size, suggesting that this meta-analysis had a certain robustness and reliability.

### 3.4. Subgroup Analyses and Metaregression Analysis

A multivariate metaregression was conducted to explore the potential factors of the heterogeneity in the pooled estimate, and results are summarized in Table 2, which demonstrated that ethnicity with *P* = 0.011 might explain the heterogeneity among these publications. Meanwhile, as presented in Table 3 for the subgroup analyses, no statistical relations were found between occupational dust exposure and mortality risk of lung cancer among different outcomes including PMR, SMR, OR, and RR (*P* > 0.05). In the subgroup of the ethnicity, the pooled estimate for the Asian population was 4.94 (3.95-6.17) with *P* < 0.001; however, exposure to dust might not increase the mortality risk of lung cancer in the Caucasian and Negro population. When compared with the general population, coalminers exposed to occupational dust would be susceptible to the mortality of lung cancer with a risk estimate of 1.23 (1.08-1.40). In addition, coalminers who worked during 1980 and 2000 obtained a higher risk (1.56, 1.10-2.20) compared to the coalminers before 1980 and after 2000. In the cohort studies, coalminers were more susceptible to death from lung cancer with a relative risk of 1.42 (1.10-1.85). Studies from China indicated a higher risk for coalminers with RR of 4.94 (3.95-6.17). For miners with duration of follow-up less than 10 years, the mortality risk was 1.26 (1.00-1.56). In addition, there was a statistical significant correlation between dust exposure and lung cancer mortality with the relative risk of 1.28 (1.07-1.53) in the studies with high quality. But such a correlation was not found in the studies with moderate quality. With respect to the other types of subgroup analyses including the case and control groups, types of the study design, and observed deaths, there were no statistical significant correlations between exposure to occupational dust and mortality risk of lung cancer.

Overall, these results suggested that exposure to occupational dust would significantly increase the mortality risk of lung cancer, especially for Asian population in China, which prompted that relevant departments should take more strict measures to measure and control occupational dust and strengthen the occupational protection and health promotion for coalminers.

## 4. Discussion

Although a global movement toward alternative energy has been taken, coal still undertakes nearly a third of worldwide energy supply [27], and this continuous reliance on coal for energy will continue to keep coalminers at risk for the development of a spectrum of respiratory diseases associated with dust exposure. Despite numerous epidemiological studies being conducted, it is still debated whether exposure to coalmine dust increases the risk of lung cancer mortality. Some authors found that compared to the general population, the death risk of lung cancer for coalminers was just similar or slightly increased [14, 28, 29]. Research conducted by Tomášková et al. showed a high mortality risk of lung cancer (SMR = 1.70) for Czech coalminers compared with the general population in the period of 1992–2013 [26]. In addition, the work of Une et al. [30] and Miller with his colleagues [6, 25] also highlighted a possible relationship between long-term exposure to dust in coalmines and the mortality risk of lung cancer. However, research conducted by Goldman and Ames et al. [11, 17] indicated a decreased risk for coalminers who are exposed to the occupational coalmine dust with SMR of 0.81 and 0.89, respectively. Therefore, there is an urgent need for the accurate estimate of mortality risk from lung cancer for coalminers.

Overall, this present meta-analysis, which combined the results from 22 different studies, supports the carcinogenicity of coalmine dust on the lung with the pooled risk estimate of 1.16 (95% CI: 1.03-1.30). However, there was a significant heterogeneity among these enrolled researches (*I*^2^ = 95%, *P* < 0.001). Therefore, we conducted the sensitivity analysis and metaregression analysis to find the potential heterogeneity sources, and results indicated that the high heterogeneity might result from the different ethnicities. Different risks were also observed among different ethnicities in the following subgroup analysis. The Asian populations in China tended to be susceptible to lung cancer mortality with a pooled risk estimate of 4.94 (3.95-6.17), while this association was not found for Caucasian or Negro populations. This risk difference might result from different data sources, statistical methods, and working conditions. Since publications for the Asian populations were conducted in China from 1988 to 1994, which was a period with rapid economic development due to the implementation and progress of reform and opening up. Great progress and rapid development have been made in industrial production, especially in the coal mining industry. However, the working conditions, monitoring, and control of pollutants as well as the awareness of own protection for coalminers have not been improved accordingly. Therefore, the morbidity and mortality risk of various respiratory diseases rose considerably among coalminers. Based on subgroup analysis for the year of publication, we observed an excessive mortality risk of 1.56 (1.10-2.20) from 1980 to 2000. In addition, there was a statistical significant correlation between dust exposure and lung cancer mortality with the relative risk of 1.42 (1.10-1.85) in cohort studies, especially in studies with high quality (RR: 1.28, 1.07-1.53). Result from the subgroup analysis for Asian populations from China can be strongly convincing since no significant heterogeneity existed (*I*^2^ = 39.3; *P* = 0.192). However, there was high heterogeneity in the data synthesis for both overall and all other subgroup analysis with *I*^2^ > 50% and *P* < 0.05, so these results should be interpreted with caution by the relevant groups and researches.

Strengths of our meta-analysis included its comprehensive and up-to-date search for both the electronic databases and reference lists of the enrolled publications. In addition, more than 8909 events from varied areas, ethnicities, and study designs were enrolled in this analysis, which can decrease the risk of bias overall to provide a more convincing and generic conclusion.

Despite the obvious strengths, some limitations still existed in this study. Firstly, limitations of this study included the variation in study settings and individual participants, which might cause the high heterogeneity among these publications. In addition, publications without complete information at individual participant level or study level were removed in our meta-analysis, resulting in a limited number of enrolled studies and sample size. Although metaregression and subgroup analyses were performed, the pooled estimates should be interpreted cautiously since there is high heterogeneity among these publications. Therefore, prospective large cohort studies are warranted to verify the results of this study that were attained through pooled analysis of 22 studies conducted in varied protocols and settings.

To conclude, this systematic review and meta-analysis supports the positive association between coalmine dust and mortality risk of lung cancer, especially for Asian populations from China when compared to the general population during 1980 and 2000. The magnitude of this effect is of major global public health importance in view of the ubiquitous existence of the coalmining industry worldwide, which should prompt relevant departments to take more strict measures for the measurement and control of the occupational dust and the protection for coalminers.

## Figures and Tables

**Table 1:** The main features of 22 enrolled studies in this meta-analysis.

Study	Year	Area	Observed deaths	Duration of follow-up (years)	Outcome type	Outcome with 95% confidence interval	Control group	Case group	Type of research	Quality assessment
Ames RG	1983	US	NA	16	OR	0.89 (0.66, 1.20)	Coalminers	Coalminers	CC	8
Attfield MD	2008	US	331	2	SMR	1.07 (0.95, 1.19)	GP	Coalminers	DS	9
Boyd JT	1970	UK	39	19	PMR	0.84 (0.60, 1.15)	GP	Coalminers	DS	8
Coggon D	1995	UK	4610	9	PMR	0.92 (0.89, 0.94)	GP	Coalminers	DS	6
Costello J	1974	US	24	9	SMR	0.67 (0.43, 0.99)	GP	Coalminers	DS	7
Enterline PE	1964	US	161	1	SMR	1.92 (1.63, 2.24)	GP	Coalminers	DS	6
Enterline PE	1972	US	4	25	SMR	1.11 (0.30, 2.85)	GP	Coalminers	DS	8
Goldman KP	1965	UK	30	1	SMR	0.81 (0.55, 1.16)	GP	Coalminers	DS	5
Goldman KP	1965	UK	54	1	SMR	0.92 (0.69, 1.19)	Quarrymen	Coalminers	DS	5
Goldman KP	1965	UK	216	5	SMR	0.70 (0.61, 0.80)	Quarrymen	Coalminers	DS	5
Graber JM	2014	US	568	38	SMR	1.08 (1.00, 1.18)	GP	Coalminers	CS	9
Guo P	1994	China	61	9	SMR	5.56 (4.26, 7.15)	GP	CM	CS	8
Hrubec Z	1995	US	26	26	RR	1.30 (0.91, 1.74)	GP	Coalminers	DS	7
Kuempel ED	1995	US	65	10	SMR	0.77 (0.60, 0.99)	Coalminers	Coalminers	CS	9
Miller BG	2009	UK	958	21	SMR	0.99 (0.93, 1.05)	GP	Coalminers	CS	9
OPCS	1978	UK	843	2	PMR	1.15 (1.07, 1.23)	GP	Coalminers	DS	6
Rockette	1977	US	352	12	SMR	1.13 (1.02, 1.26)	GP	Coalminers	CS	9
Swaen GMH	1995	Netherlands	272	37	SMR	1.02 (0.90, 1.12)	GP	CM	CC	7
Tomášková H	2017	Czech	116	21	SMR	1.70 (1.41, 2.04)	GP	CM	CS	8
Tomášková H	2017	Czech	143	21	SMR	0.83 (0.70, 0.98)	GP	Coalminers	CS	9
Wang JW	1988	China	36	11	RR	3.69 (2.00, 6.78)	GP	Coalminers	CS	9
Wang ZG	1992	China	NA	9	OR	3.24 (1.69, 6.20)	Coalminers	Coalminers	CC	8

Abbreviation: CC: case control study; CM: coalminers with pneumoconiosis; CS: cohort study; DS: descriptive study; OPCS: Office of Population Censuses and Surveys; OR: odds ratio; PMR: proportional mortality ratio; RR: relative risk; SMR: standardized mortality ratio.

**Table 2:** Results of metaregression analysis.

Parameter	Coefficient	95% CI	*P*
Year of publication	0.232	-0.125, 0.590	0.182
Outcome type	0.042	-0.216, 0.230	0.730
Ethnicity	-0.767	-1.325, -0.208	**0.011**
Control group	0.203	-0.109, 0.515	0.182
Case group	0.226	-0.465, 0.917	0.489
Type of research	0.172	-0.126, 0.470	0.233
Observed deaths	-0.143	-0.591, 0.305	0.501
Area	0.071	0.352, 0.494	0.722
Duration of follow-up	-0.310	-1.385, 2.452	0.103

Abbreviation: 95% CI: 95% confidence interval.

**Table 3:** Results of subgroup analyses for the selected studies.

Prespecified or not	Groups	Subgroups	No. of studies	No. of events	Outcome with 95% CI	*P* value for outcome	*I* ^2^ (%)	*P* value for heterogeneity
Yes	Outcome type	SMR	15	3355	1.13 (0.95-1.35)	0.154	95.3	<0.001
		PMR	3	5492	0.99 (0.82-1.20)	0.901	94.2	<0.001
		OR	2	NA	1.64 (0.46-5.82)	0.442	92.0	<0.001
		RR	2	62	2.12 (0.76-5.88)	0.149	88.6	0.003

Yes	Ethnicity	Asian	3	97	**4.94 (3.95-6.17)**	**<0.001**	39.3	0.192
		Caucasian	15	7824	1.05 (0.94-1.17)	0.402	92.4	<0.001
		Caucasian/Negro	4	988	0.96 (0.82-1.12)	0.595	72.3	0.013

No	Control group	General population	17	8574	**1.23 (1.08-1.40)**	**0.002**	95.5	<0.001
		Coalminers	3	65	1.19 (0.66-2.15)	0.558	87.8	<0.001
		Quarrymen	2	270	0.78 (0.60-1.02)	0.066	67.7	0.078

No	Case group	Coalminers with pneumoconiosis	3	449	2.11 (0.89-5.03)	0.091	98.6	<0.001
		Coalminers	19	8460	1.04 (0.94-1.14)	0.472	90.8	<0.001

Yes	Type of research	Cohort study	8	2299	**1.42 (1.10-1.85)**	**0.008**	96.8	<0.001
		Case control study	3	272	1.26 (0.81-1.96)	0.312	84.5	0.002
		Descriptive study	11	6338	1.01 (0.87-1.17)	0.933	92.9	<0.001

No	Observed deaths	<100	9	339	1.28 (0.74-2.22)	0.376	95.5	<0.001
		>100	13	8570	1.10 (0.99-1.22)	0.086	94.0	<0.001

No	Year of publication	1964-1980	9	1723	0.99 (0.80-1.23)	0.935	92.3	<0.001
		1980-2000	8	5070	**1.56 (1.10-2.20)**	**0.012**	96.9	<0.001
		2000-2017	5	2116	1.09 (0.94-1.26)	0.247	89.4	<0.001

No	Area	China	3	97	**4.94 (3.95-6.17)**	**<0.001**	39.3	0.192
		UK	7	6750	0.92 (0.82-1.03)	0.137	89.4	<0.001
		US	9	1531	1.09 (0.92-1.29)	0.338	87.3	<0.001
		Others	3	531	1.13 (0.79-1.61)	0.521	94.1	<0.001

No	Duration of follow-up (years)	<10	10	6330	**1.26 (1.00-1.56)**	**0.048**	97.2	<0.001
		>10 and <20	5	492	1.08 (0.80-1.47)	0.607	85.2	<0.001
		>20	7	2087	1.10 (0.96-1.26)	0.172	84.8	<0.001

No	Study quality	Moderate	8	5964	1.01 (0.84-1.22)	0.908	94.8	<0.001
		High	14	2945	**1.28 (1.07-1.53)**	**0.006**	94.4	<0.001

Abbreviation: OR: odds ratio; PMR: proportional mortality ratio; RR: relative risk; SMR: standardized mortality ratio; 95% CI: 95% confidence interval.

## Data Availability

The relevant data has been included in the manuscript and supplementary material.

## Abbreviations

AHRQ: Agency for Healthcare Research and Quality
CNKI: Chinese National Knowledge Infrastructure
IARC: International Agency for Research on Cancer
NOS: Newcastle-Ottawa Scale
OR: Odds ratio
PMR: Proportional mortality ratio
PRISMA: Preferred Reporting Items for Systematic Reviews and Meta-Analyses
RR: Relative risk
SMR: Standardized mortality ratio
95% CI: 95% confidence intervals.

## Supplementary Materials

Supplementary Materials Supplementary material shows the detailed results of the assessment of quality and bias risk of the descriptive studies (Table S1), case control studies (Table S2), and cohort studies (Table S3). The methodological quality of the descriptive studies was assessed using the 11-item checklist recommended by the Agency for Healthcare Research and Quality (AHRQ), while the Newcastle-Ottawa Scale (NOS) was followed for the assessment of quality and bias risk for case control studies and cohort studies. An item would be scored “0” if it was answered “UNCLEAR” or “NO”; for the answer of “YES”, the item would get a “1” score or “2” score (only for comparability between groups).
