# Supplementary material for: Association between Coalmine Dust and Mortality Risk of Lung Cancer: A Meta-Analysis
Source: Biomed Res Int. 2021 Mar 8;2021:6624799. doi: 10.1155/2021/6624799 (PMC7963907; doi:10.1155/2021/6624799)
Supplement: Supplementary Materials — Supplementary material shows the detailed results of the assessment of quality and bias risk of the descriptive studies (Table S1), case control studies (Table S2), and cohort studies (Table S3). The methodological quality of the descriptive studies was assessed using the 11-item checklist recommended by the Agency for Healthcare Research and Quality (AHRQ), while the Newcastle-Ottawa Scale (NOS) was followed for the assessment of quality and bias risk for case control studies and cohort studies. An item would be scored “0” if it was answered “UNCLEAR” or “NO”; for the answer of “YES”, the item would get a “1” score or “2” score (only for comparability between groups). [file 6624799.f1.pdf]

Association between coalmine dust and mortality risk of lung cancer: a meta analysis

Linlin Li [1,2] , Min Jiang [3], Xuelian Li [3] and Baosen Zhou [1*]

[1] Department of Clinical Epidemiology, First Affiliated Hospital, China Medical University, Shenyang 110001, China.

[2] Medical Oncology Department of Thoracic Cancer (2), Cancer Hospital of China Medical University, Liaoning Cancer Hospital & Institute, No.44, Xiaoheyan Road, Dadong District, Shenyang 110042, Liaoning Province, People's Republic of China

[3] Department of Epidemiology, School of Public Health, China Medical University, Shenyang 110122, China.

13504982940@163.com (L.L.); mij9620@163.com (M.J.); xlli@cmu.edu.cn (X.L.); bszhou@cmu.edu.cn (B.Z.).

[*] Corresponding Author: Baosen Zhou

Address: No.155 Nanjing Bei Street, Shenyang, Liaoning 110001, People's Republic of China.

Tel.: +86-13386881563; E-mail Address: bszhou@cmu.edu.cn

Table S1 Assessment of study quality and risk of bias for cross-sectional study

Research	Define the source of information（1）	List inclusion and exclusion criteria for exposed and unexposed subjects （1）	Indicate time period used for identifying patients （1）	Indicate whether or not subjects were consecutive （1）	Explain any patient exclusions from analysis （1）	Indicate if evaluators of subjective components of study were masked to other aspects of the status of the participants （1）	Describe any assessments undertaken for quality assurance purposes（1）	Describe how confounding was assessed and/or controlled （1）	Explain how missing data were handled in the analysis （1）	Summarize patient response rates and completeness of data collection（1）	Clarify what follow-up and the percentage of patients for which incomplete data or follow-up was obtained （1）	Total score（11）
Coggon D 1995	1	1	1	1	1	1	0	0	0	0	0	6
Enterline PE 1964	1	1	1	1	1	1	0	0	0	0	0	6
Hrubec Z 1995	1	1	1	1	1	1	0	1	0	0	0	7
Office of Population Censuses and Surveys 1978	1	1	1	1	1	1	0	0	0	0	0	6

Table S2 Assessment of study quality and risk of bias for case control study

Research	Is the case definition adequate	Representativeness of the cases	Selection of Controls	Definition of Controls	Comparability of cases and controls on the basis of the design or analysis	Ascertainment of exposure	Same method of ascertainment for cases and controls	Non-Response rate	Total scores（9）
Wang ZG 1992	1	1	1	1	2	1	1	0	8
Ames RG 1983	1	1	1	1	2	0	1	1	8
Swaen GMH 1995	0	0	1	1	2	1	1	1	7

Table S3 Assessment of study quality and risk of bias for cohort study

Research	Representativeness of the exposed cohort（1）	Selection of the non-exposed cohort（1）	Ascertainment of exposure（1）	Demonstration that outcome of interest was not present at start of study（1）	Comparability of cohorts on the basis of the design or analysis（2）	Assessment of outcome（1）	Was follow-up long enough for outcomes to occur（1）	Adequacy of follow up of cohorts（1）	Total scores（9）
Rockette 1977	1	1	1	1	2	1	1	1	9
Wang JW 1988	1	1	1	1	2	1	1	1	9
Guo P 1994	1	1	1	1	2	1	0	1	8
Kuempel	1	1	1	1	2	1	1	1	9

ED 1995									
Miller BG 2009	1	1	1	1	2	1	1	1	9
Graber JM 2014	1	1	1	1	2	1	1	1	9
Tomášková H 2017	1	1	1	1	2	1	1	1	9
Tomášková H 2017	0	1	1	1	2	1	1	1	8
Attfield MD 2008	1	1	1	1	2	1	1	1	9
Boyd JT 1970	1	1	1	1	1	1	1	1	8
Costello J 1974	1	1	1	1	1	1	1	1	8
Enterline PE 1972	1	1	1	1	1	1	1	1	8
Goldman KP 1965	1	1	1	1	1	0	1	1	7
Goldman KP 1965	1	1	1	1	1	0	1	1	7
Goldman KP 1965	1	1	1	1	1	0	1	1	7
